# On-column refolding and off-column assembly of parvovirus B19 virus-like particles from bacteria-expressed protein

**DOI:** 10.1007/s00253-024-13004-w

**Published:** 2024-01-22

**Authors:** Ignacio Sánchez-Moguel, Carlos Francisco Coffeen, Ismael Bustos-Jaimes

**Affiliations:** Departamento de Bioquímica, Facultad de Medicina, Universidad Nacional Autónoma de México, 04510 Mexico City, Mexico

**Keywords:** Parvovirus B19, Virus-like particles, Self-assembly, Protein refolding, Off-column assembly

## Abstract

**Abstract:**

Virus-like particles (VLPs) are nanometric structures composed of structural components of virions, keeping most of the cellular recognition and internalization properties, but are non-infective as they are deprived of their genetic material. VLPs have been a versatile platform for developing vaccines by carrying their own or heterologous antigenic epitopes. Moreover, VLPs can also be used as nanovessels for encapsulating molecules with therapeutic applications, like enzymes, nucleic acids, and drugs. Parvovirus B19 (B19V) VLPs can be self-assembled in vitro from the denatured major viral particle protein VP2 by equilibrium dialysis. Despite its fair productivity, this process is currently a time-consuming task. Affinity chromatography is used as an efficient step for concentration and purification, but it is only sometimes seen as a method that facilitates the oligomerization of proteins. In this research, we report a novel approach for the in vitro assembly of B19V VLPs through the immobilization of the denatured VP2 into an immobilized metal affinity chromatography (IMAC) column, followed by the on-column folding and the final VLP assembly upon protein elution. This method is suitable for the fast production of B19V VLPs.

**Key points:**

*• Biotechnological applications for inclusion bodies*

*• Efficient single-step purification and immobilization strategies*

*• Rapid VLP assembly strategy*

**Graphical abstract:**

**Figure:**
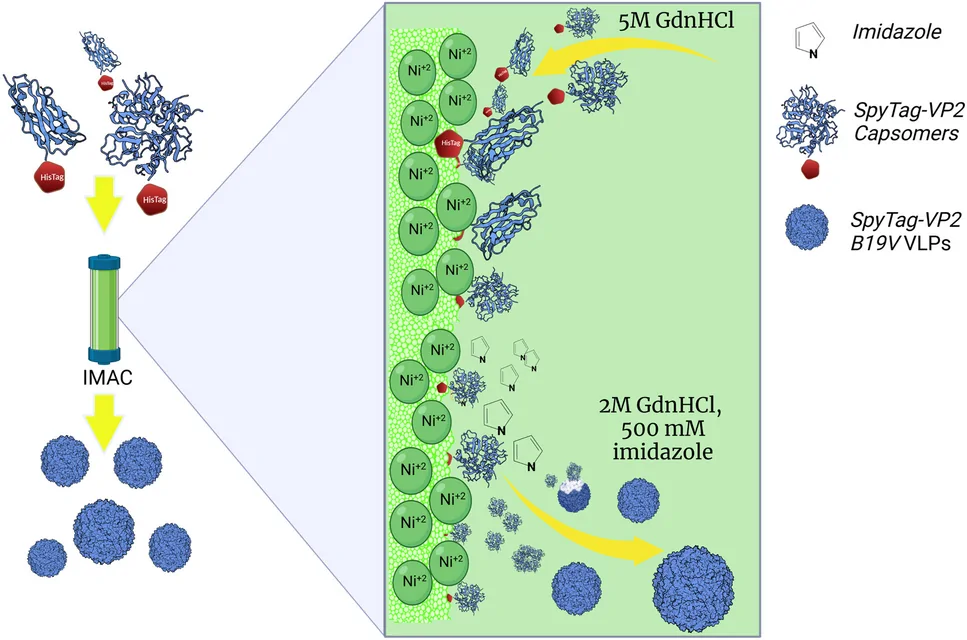

## Introduction

Virus-like particles (VLPs) are assemblies of viral proteins, excluding their genetic material. The lack of infectivity and the size in the nanometric scale of VLPs have attracted the pharmaceutical industry’s attention for applications ranging from developing vaccines to designing tissue-specific drug-delivery vectors (Loo et al. 2021). Fully assembled VLPs can be produced in prokaryotic and eukaryotic expression systems. Nevertheless, some systems need to be more productive for industrial manufacture and may be subjected to application limitations imposed by the expression system. The main limitation is the chemical definition of the VLPs, as the production of VLPs in any cell is prone to include undesirable molecules inside the particles. Therefore, further disassembly, purification, and reassembly may be required before application (Le and Müller 2021). In contrast, recombinant structural viral proteins can be produced in high yield into suitable expression systems and then purified and assembled in controlled conditions. High-level expression of recombinant proteins in bacteria often results in the formation of inclusion bodies (IBs), insoluble protein aggregates that demand an appropriate strategy for their solubilization and refolding.

Our group has expressed the major structural protein (VP2) of the parvovirus B19 (B19V) capsid in *E. coli*. This protein is produced as IBs and then resuspended in 6 M guanidinium hydrochloride (GdnHCl) and purified by IMAC. The resulting protein can be self-assembled into VLPs by removing the GdnHCl through equilibrium dialysis against PBS added with 0.2 M L-Arginine (PBS-Arg) for 48 h (Sánchez-Rodríguez et al. 2016). Dialysis is a time-consuming process with few industrial applications, while the directed dilution of the denatured protein is fast but produces a large amount of misfolded protein. To reduce the time-consuming dialysis step, we explored removing the chaotropic agent by another procedure than dialysis, like on-column buffer exchange (Lemercier et al. 2003; Oganesyan et al. 2005). While the Tag-immobilization approaches have been mainly explored for independent protein purification, efficient strategies combining Tag-mediated purification and immobilization/functionalization/oligomerization are advantageous for practical applications (Freitas et al. 2022). The reports of strategies for obtaining refolded proteins through chromatographic techniques and affinity Tags to recover functional proteins able to form supramolecular structures like VLPs are scarce. In this research, we hypothesized that binding the unfolded VP2 to an IMAC column followed by a buffer exchange with a lower amount of the chaotropic agent would allow the refolding of the monomer without assembly until monomers were released from the column upon elution. Our results showed that this method makes it possible to produce B19V VLPs. This technique, which we named “off-column assembly,” is a promising new approach to enhance the production of VLPs at an industrial scale and expands the potential applications of these particles as vaccine platforms and drug delivery systems.

## Materials and methods

### Chemicals

Isopropyl-β-D-thiogalactoside (IPTG) was purchased from GoldBio U.S.A. GdnHCl, L-Arginine, Glycine, NaCl, NaH_2_PO_4_, Tris–HCl, ethylenediaminetetraacetic acid (EDTA), phenylmethanesulfonyl fluoride (PMSF), MgSO_4_•7H_2_O, Triton X-100, Congo Red (CR), acetic acid, uranyl acetate, and imidazole were of analytical grade and were purchased from Sigma-Aldrich, Mexico. Benzonase nuclease was also purchased from Sigma-Aldrich, Mexico. Proteins for gel filtration calibration were purchased from Cytiva.

### Plasmids and strains

The plasmids pET22b( +)-SpyTag-VP2 and pET22b( +)-sfGFP-SpyCatcher, harboring genes coding for the chimeras SpyTag-VP2 and sfGFP-SpyCatcher, respectively, were previously constructed in our research group (Cayetano-Cruz et al. 2018, 2019). Plasmids were transferred into *E. coli* BL21(DE3) by the CaCl_2_ and the heat shock method.

### Protein expression and purification

**Fig. 1:**
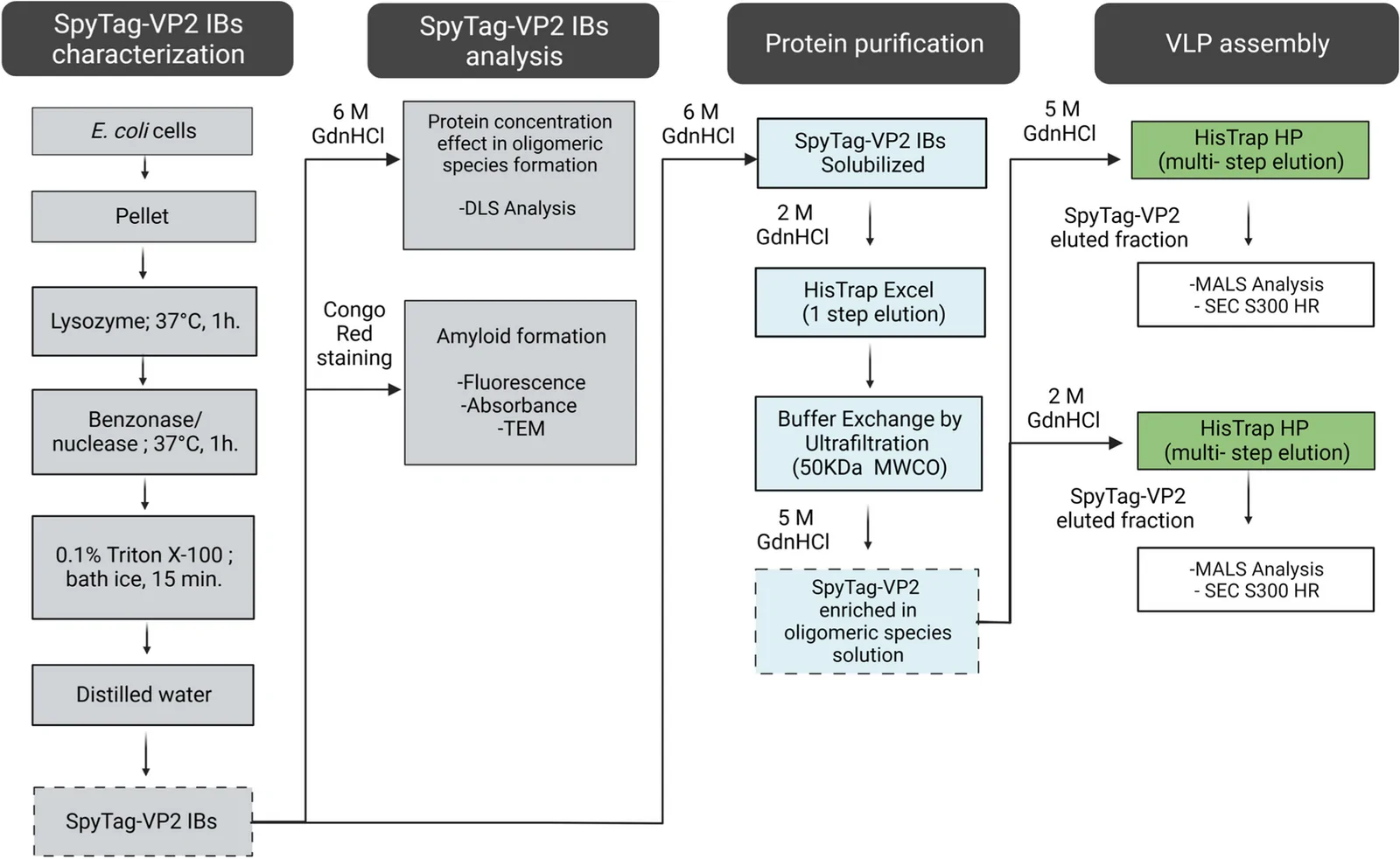
Flow diagram of the methodology

Here, we used the previously reported N-terminal SpyTag chimera of VP2 (SpyTag-VP2) as the model protein for VLP assembly. The sfGFP-SpyCatcher chimera was also produced to test the functionality of the SpyTag peptide present in the produced SpyTag-VP2 VLPs. Chimeric proteins SpyTag-VP2 and sfGFP-SpyCatcher were expressed in *E. coli* and purified by IMAC as previously described (Cayetano-Cruz et al. 2018, 2019) and resumed in Fig. 1.

### SpyTag-VP2 IB characterization

#### IB production and purification

SpyTag-VP2 as well as other N-terminal chimeras of VP2, expressed in bacteria, accumulates in the form of IBs. The high concentration of GdnHCl required to solubilize the IBs from VP2 chimeras suggests that they may have amyloid nature. Here, we analyzed this possibility by purification of IBs and spectroscopic analysis. The cells were disrupted by sonication, and the insoluble fraction was recovered by centrifugation and resuspended in a lysis buffer containing 50 mM Tris–HCl pH 8.0, 100 mM NaCl, and 1 mM EDTA; 1 mL of suspension was added with 10 µL PMSF (100 mM) and 50 µL lysozyme (10 mg/mL) and incubated for 1 h at 37 °C under shaking conditions. The sample was incubated on ice for 1 h, centrifuged, and washed with lysis buffer. Then, 0.5 µL of benzonase nuclease (250 UI/µL) and 50 µL of MgSO_4_ (1 M) were added to the sample and incubated for 1 h at 37 °C under shaking conditions. The samples were centrifuged and washed with lysis buffer containing 0.5% (v/v) Triton X-100 and incubated in ice for 15 min. The pellets were resuspended in 1 mL of distilled water and washed twice. The IB suspension obtained was frozen at − 20 °C until its use. The SpyTag-VP2 protein concentration in IB suspension was determined by densitometry using ImageJ Software.

#### Congo Red staining of IBs

The amyloid specific dye Congo Red (CR) was used to detect typical β-sheet structures in amyloid aggregates. The staining was carried out through a modification of a previously reported method (Klunk et al. 1989). The purified IBs containing SpyTag-VP2 were resuspended in 1 mL of distilled water; 60 µL of IB sample was added to 440 µL of a solution containing 20 µM Congo Red (CR), 5 mM sodium phosphate buffer, and 150 mM NaCl, pH 7.4. The samples were incubated overnight at 4 °C, and 200-µL samples were dispensed in a 96-well plate, and the optical absorption spectra were acquired from 400 to 700 nm using a Polarstar Omega plate reader (BMG Labtech). The emission fluorescence spectra were acquired from 550 to 750 nm with an excitation wavelength of 520 nm in an ISS PC1 Spectrofluorometer (Champaign IL-USA).

#### Effect of protein concentration on oligomeric species formation

The diameter of the soluble or suspended species of the SpyTag-VP2 IBs in 6 M GdnHCl was studied by DLS. Samples of 5, 10, 15, 20, and 25 µL of IB suspension (1.2 mg/mL of SpyTag-VP2) were diluted in 1 mL of solubilization buffer (6 M GdnHCl, 0.3 M NaCl, 50 mM sodium phosphate, pH 6.3) and then filtered through 0.22-µm filter membrane. DLS analysis was performed in a Zetasizer Ultra (Malvern Panalytical), setting the lecture at the forward angle (13°), using a glass cuvette with 400 µL of the sample. The measurements were performed with 240 s of stabilization time at 25 °C.

### SpyTag-VP2 VLP on-column refolding and off-column assembly

#### Protein concentration

A first step of concentration and partial purification of the protein was carried out by IMAC. SpyTag-VP2 IBs from a 1500 mL of *E. coli* BL21(DE3) culture were resuspended in 50 mL of solubilization buffer as described elsewhere (Cayetano-Cruz et al. 2018). Then, batches of 5 mL of the solubilized IBs were loaded manually into a 5 mL HisTrap Excel column (Cytiva) equilibrated with equilibration buffer (2 M GdnHCl, 0.3 M NaCl, 50 mM sodium phosphate, pH 7.4), washed with 3 column volumes of equilibration buffer, and eluted with 5 column volumes of elution buffer (equilibration buffer + 500 mM imidazole). Eluted protein batches were concentrated, and the buffer was exchanged for storage buffer (5 M GdnHCl, 0.3 M NaCl, 50 mM Tris–HCl, pH 8.0) using Amicon centrifugal filters (MWCO 50 kDa, Millipore).

#### Protein purification coupled to in-column refolding and off-column assembly of VLPs

VP2 chimeras can be purified under non-native conditions by IMAC. Here, to test the ability of the IMAC column to allow the refolding of the bound protein but not its self-assembly, the amount of GdnHCl in the binding and elution buffers was reduced to a concentration that, off-column, promotes the refolding and partial self-assembly of VLPs. Two batches of the protein in Storage Buffer at the same protein concentration (0.14 mg/mL) were prepared to study the elution behavior of SpyTag-VP2 at different concentrations of GdnHCl. The first protein batch was loaded into a 5 mL HisTrap HP column equilibrated with 5 M GdnHCl Buffer (5 M GdnHCl, 0.3 M NaCl, 50 mM sodium phosphate, pH 7.4) and eluted with the same buffer added with 500 mM imidazole. The second batch was loaded into an identical column, equilibrated with 2 M GdnHCl buffer (2 M GdnHCl, 0.3 M NaCl, 50 mM sodium phosphate, pH 7.4), and eluted with the same buffer added with 500 mM imidazole. A step gradient of 75, 150, 200, and 500 mM of imidazole at 2.5 mL/min was used to elute the proteins in 5-mL fractions using an FPLC system (Äkta Purifier, GE Healthcare). The eluted fractions were analyzed by SDS-PAGE and Multi-Angle Light Scattering (MALS) (Zetasizer Ultra, Malvern Panalytical).

#### Size exclusion chromatography

Size exclusion chromatography (SEC) allows to separate molecules by its hydrodynamic diameter, then it was applied as a preparative purification method. The eluted peaks containing SpyTag-VP2 protein were passed through a size exclusion column Sephacryl 300-HR (Cytiva) 37 mL packed column, equilibrated in 25 mM sodium acetate, 0.2 M Glycine, pH 4.0 buffer, 0.25 mL/min, using an FPLC system (Äkta Purifier, GE Healthcare). The samples collected were analyzed by MALS (Zetasizer Ultra, Malvern Panalytical). Molecular weight calibration proteins (Blue dextran 2000, thyroglobulin, ferritin, β-amylase, aldolase, ovalbumin, myoglobin, carbonic anhydrase, ribonuclease, and vitamin B_12_) were used to calibrate the column using 1 × PBS as mobile phase. Then, the hydrodynamic diameter (*D*_H_) of the SpyTag-VP2 was determined.

#### Effect of the protein concentration loaded into the IMAC column on VLP formation

In oligomeric proteins, monomer crowding is critical for the oligomerization steps involved in reaching its final oligomeric sate. This is of fundamental importance in viral proteins forming capsomers, oligocapsomers, and finally, capsids. To test the effect of crowding, different amounts of protein (2.5, 5, 10, 40, and 60 mL of a 0.142 mg/mL of SpyTag-VP2 protein) were loaded into the IMAC 5-mL HisTrap HP column (Cytiva) and eluted under the same conditions described before. Then, 2.5 mL of the SpyTag-VP2 peak fraction eluted from the HisTrap HP column was loaded into a 37-mL Sephacryl 300-HR column (Cytiva) and eluted in 2 M GdnHCl Buffer. The particle size of the SEC-eluted fractions was analyzed by MALS (Zetasizer Ultra, Malvern Panalytical). The fractions displaying species with diameters close to that of VLPs (18–28 nm) were pooled, and the protein concentration was determined by the BCA method.

### Characterization of the off-column assembled VLPs

#### Fluorescence spectroscopy

Intrinsic fluorescence of Trp residues is widely used as a probe for changes in tertiary and secondary structures of proteins. In this research, changes in the intrinsic fluorescence spectra were used to assess the effect of the denaturing agent GdnHCl on the structure of SpyTag-VP2 VLPs. Intrinsic fluorescence emission spectra of the samples were obtained at room temperature employing PC1 ISS Spectrofluorometer (Champaign IL-USA). The intrinsic fluorescence emission spectra (*λ*_exc_ 280 nm) were collected at a protein concentration of 0.5 mg/mL, whereas the emission was monitored between 300 and 450 nm using excitation and emission slit widths of 1 mm. Spectra were smoothed using the instrument software.

#### Hemagglutination of human red blood cells

B19V virions or VP2-derived VLPs can agglutinate human red blood cells (RBCs) (Bieri et al. 2021; Soto-Valerio et al. 2022); therefore, the hemagglutination (HA) reaction implies the correct folding and assembly of these particles. RBCs were washed 3 times with a sterile isotonic solution, and then, a 1% suspension in PiBS (PIPES 50 mM, NaCl 123 mM, KCl 2.7 mM, pH 6.3) was prepared. In the first well of a v-bottom well plate, 150 µL of SpyTagVP2 particles (1 mg/mL) in 25 mM acetate buffer pH 4.5 were placed, and a continuous 1:2 serial dilution was performed with PiBS. Next, 50 µL of the 1% RBC suspension was added to each well and gently mixed. The plate was incubated at room temperature for at least 1 h. HA was inspected visually, and the titer was estimated as the inverse of the highest dilution of the sample that completely inhibited the RBCs’ sedimentation.

#### Circular dichroism spectroscopy

Folded proteins have characteristic circular dichroism (CD) spectra related to their secondary structures. Here, CD analysis was used to assess the structural state of the off-column assembled SpyTag-VP2 VLPs. SEC-eluted protein fractions in 2 M GdnHCl buffer, showing the hydrodynamic radius of VLPs by DLS, were pooled. Then, the buffer was exchanged for 25 mM sodium acetate pH 4.5 buffer using a desalting G25 column, and the protein concentration was adjusted to 0.5 mg/mL. SpyTag-VP2 VLP CD spectra were obtained in a Chirascan Spectropolarimeter (Applied Photophysics) at 1-nm bandwidth, in the 200–260 nm wavelength range in a 1-mm cuvette.

#### sfGFP-SpyCatcher SpyTag-VP2 conjugation reaction

To detect the presence of the N-termini of SpyTag-VP2 on the external surface of the particles, the sfGFP-SpyCatcher was used for bioconjugation with its biorthogonal partner SpaTag. The reaction was performed by mixing the molar ratio 2:1 of the SpyCatcher/SpyTag proteins in PBS 1.5 × added with 0.4 M L-Arginine, pH 7.4, and incubated in a Thermomixer (Eppendorf) at 20 °C, 550 rpm, overnight. The sample was loaded in a Superdex 200, 23 mL column to separate the unconjugated sfGFP-SpyCatcher protein. The samples were analyzed by MALS (Zetasizer Ultra, Malvern Panalytical).

#### TEM analysis samples

The morphological analysis of the particles was carried out by transmission electron microscopy. The samples eluted from IMAC column in GndHCl buffer were diluted 1:300 in Acetate-Gly buffer (acetic acid 25 mM, glycine 50 mM, pH 4.0), filtered through a 0.22-µm membrane filter, fixed for 7 min in Formvar/carbon-coated copper grids, and negatively stained with 1% (w/v) of Uranyl acetate (pH 4.0) for 5 min.

## Results

### SpyTag-VP2 expression and IB characterization

**Fig. 2:**
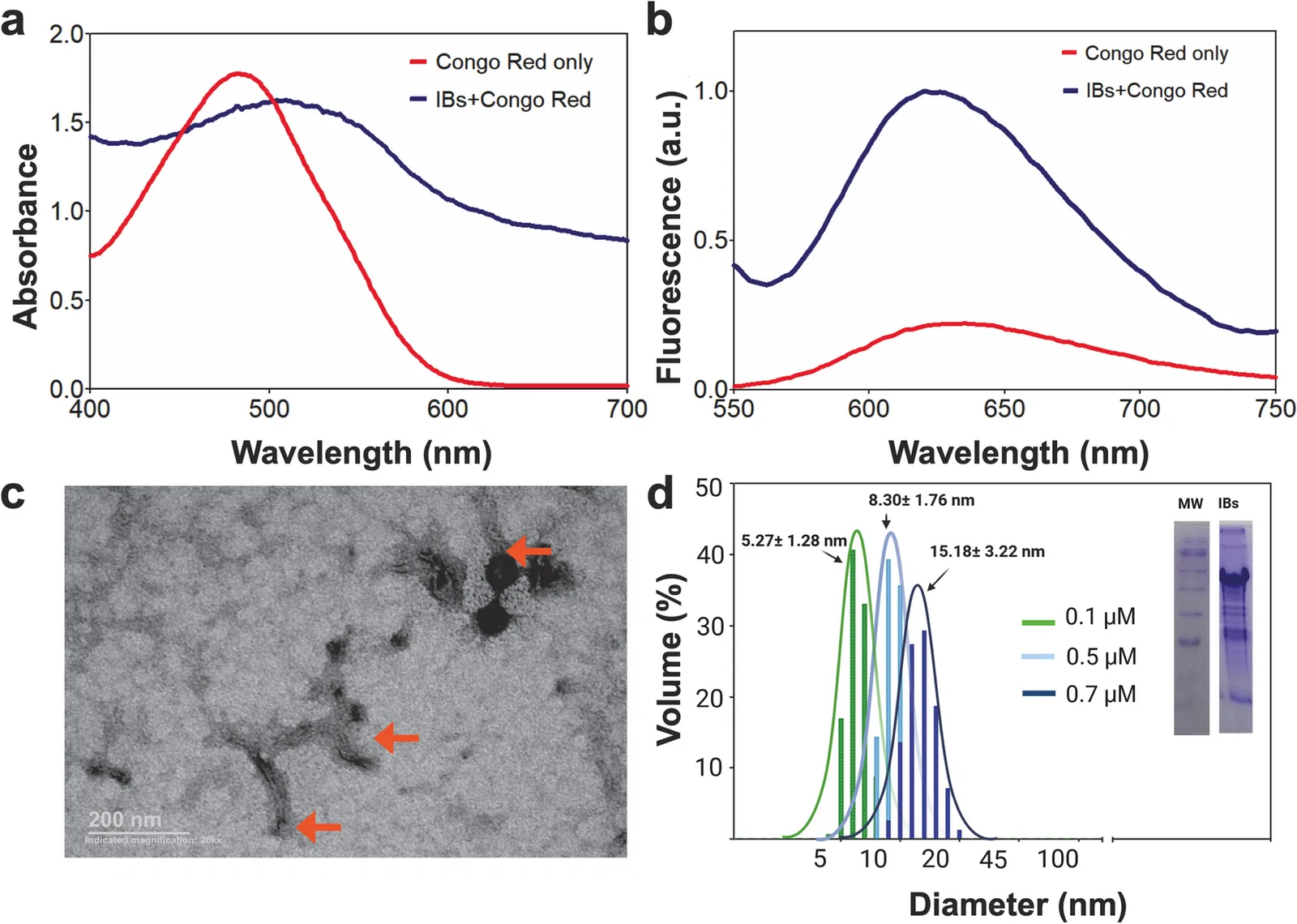
UV absorbance spectra of Congo Red and SpyTag-VP2 IBs stained with Congo Red (**a**). Fluorescence emission spectra of Congo Red and SpyTag-VP2 IBs stained with Congo Red (**b**). TEM image of SpyTag-VP2 IBs. The arrows indicate fibrillar structures (**c**). Diameters of the species eluted from the IMAC column loaded with different concentrations of SpyTag-VP2 measured by DLS (**d**)

The chimeric protein SpyTag-VP2 was expressed as IBs, then partially purified, suspended, and stained with CR. The UV spectrum of the CR-stained IBs presents the characteristic shift from 490 to 520 nm in UV absorbance observed when CR binds to amyloid fibrils (Fig. 2a). Also, the fluorescence emission analysis of the same sample revealed an increase in CR fluorescence intensity at 620 nm, typically observed when the CR binds to amyloid fibrils (Fig. 2b). Both results suggest a fibrillar structure of the IBs produced from SpyTag-VP2 (Kan et al. 2019). Accordingly, fiber-like structures resembling amyloid structures were observed by TEM of the IBs (Fig. 2c).

The SpyTag-VP2 concentration in the IB suspension (1.2 mg/mL) was estimated by densitometry using ImageJ software. Considering only the amount of SpyTag-VP2 in the sample, different amounts of IBs were solubilized in solubilization buffer to produce solutions of 0.1, 0.5, and 0.7 µM of this protein. The solutions were loaded in an IMAC column and then eluted as described in the “Materials and methods” section, and the fractions containing the SpyTag-VP2 were analyzed by DLS. Species of different hydrodynamic diameters were detected; the higher the protein concentration, the larger the species’ diameter (Fig. 2d). Considering the diameter distribution of the soluble species, it is possible that such species reflect different association states of the protein from partially denatured monomers at the low protein concentration (0.1 µM) to oligomeric forms of those monomers at concentrations of 0.5 and 0.7 µM. These oligomers, however, might play a seeding role in the latter association of the protein to form capsomers and, finally, capsids.

### SpyTag-VP2 IMAC elution profile and characterization

**Fig. 3:**
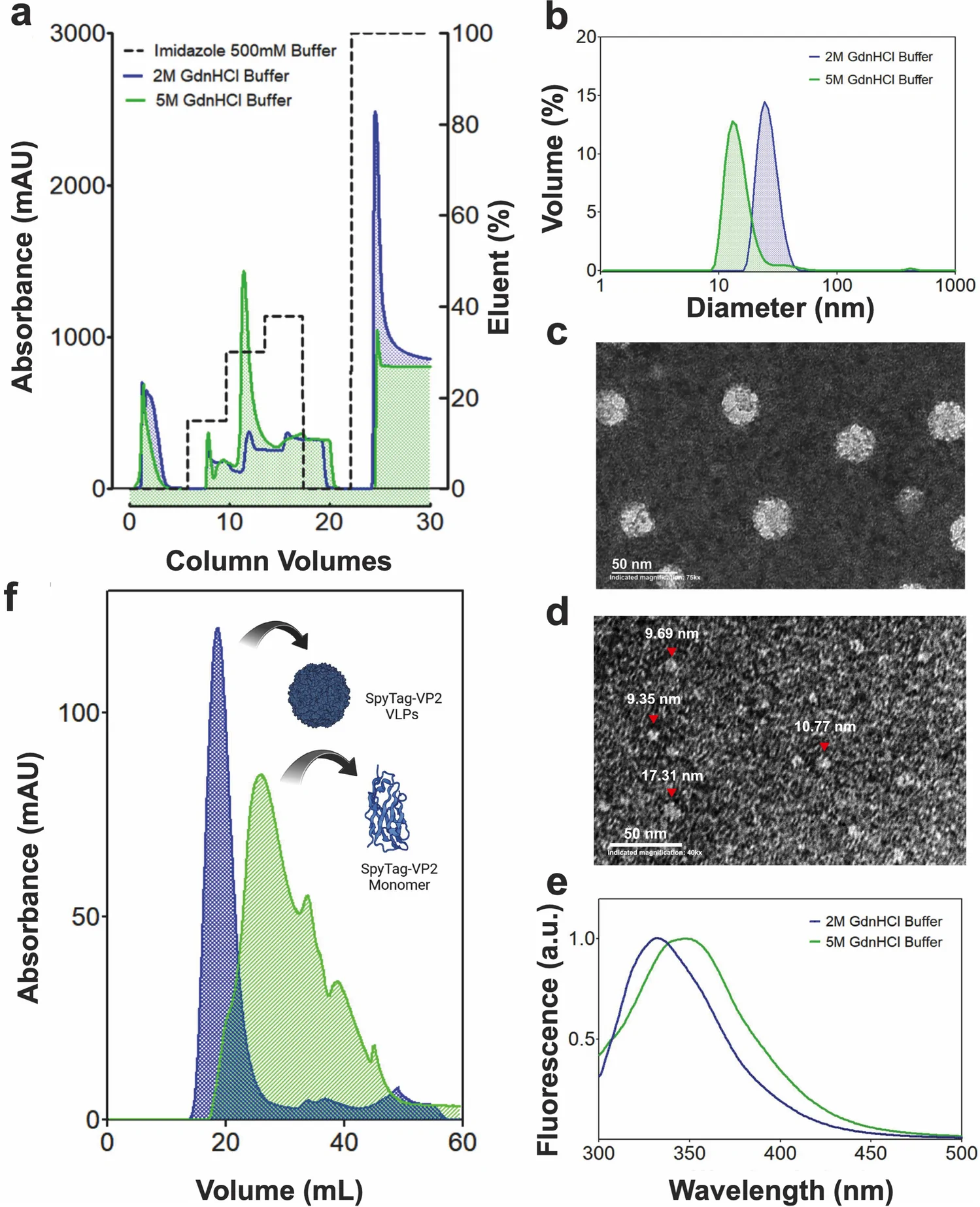
IMAC elution profiles of SpyTag-VP2 at 2 and 5 M GdnHCl from the HisTrap HP column (**a**). MALS analysis of the SpyTag-VP2 fractions eluted with 5 M GdnHCl (13 ± 4.4 nm) and 2 M GdnHCl (25 ± 5.4 nm) (**b**). TEM analysis of the peak eluted from the HisTrap HP column with 500 mM imidazole at 2 M GdnHCl (**c**). TEM analysis of the peak eluted from the HisTrap HP column with 150 mM imidazole at 5 M GdnHCl (**d**). Fluorescence emission spectra of the peak eluted from the HisTrap HP column at 2 and 5 M GdnHCl (**e**). SEC elution profiles of SpyTag-VP2 eluted at 2 and 5 M GdnHCl from the IMAC column loaded in a Sephacryl S300-HR column (**f**)

SpyTag-VP2 protein was concentrated at 0.14 mg/mL, and its off-column assembly was studied at high and mild concentrations (5 and 2 M) of the chaotropic agent GdnHCl. Initially, the concentrated protein showed a hydrodynamic diameter of 13 ± 4.4 nm, which agrees with the absence of VLPs. The protein was in a high concentration of GdnHCl and under the optimal concentration for self-assembly (0.4–0.7 mg/mL), so the presence of VLPs was not expected. Next, 60 mL protein batches were loaded into HisTrap HP columns equilibrated with buffers containing either 5 or 2 M of GdnHCl and eluted with adequate buffers with the same concentrations of GdnHCl used for binding. When the elution buffer contained 5 M GdnHCl, the SpyTag-VP2 protein eluted at 150 mM of imidazole. In contrast, when the elution buffer contained 2 M GdnHCl, the SpyTag-VP2 protein eluted at 500 mM of imidazole (Fig. 3a). The protein peak eluted with 5 M GdnHCl buffer presented the same diameter as the SpyTag-VP2 protein before column binding, as determined by MALS (13 ± 4.4 nm). Contrastingly, the SpyTag-VP2 protein eluted with 2 M GdnHCl showed a monodisperse peak of 25 ± 5.4 nm when analyzed by the same technique (Fig. 3b). TEM analysis of the SpyTag-VP2 protein eluted at 2 M GdnHCl showed the presence of VLPs (Fig. 3c), while the protein eluted at 5 M GdnHCl shows species of diameters below the expected for a B19V VLP (Fig. 3d). Their Trp intrinsic fluorescence spectra were taken to confirm the different nature of the species eluted at 5 M GdnHCl from the species eluted at 2 M GdnHCl, finding a bathochromic shift at high GdnHCl concentration that reveals a different structural state of those species (Fig. 3e). Thereby, in this approach, the assembly of the oligomers of SpyTag-VP2 is driven just by decreasing the chaotropic agent concentration from 5 to 2 M of GdnHCl in the same buffer (50 mM phosphate, 300 mM NaCl, pH 7.4).

### SpyTag-VP2 size exclusion characterization

SpyTag-VP2 protein eluted at concentrations of 5 or 2 M GdnHCl were analyzed by SEC in a Sephacryl S300-HR column. The protein fraction obtained by elution in 5 M GdnHCl showed an exclusion volume corresponding to the hydrodynamic diameter of a monomer (D_H_ of 5.8 nm) in a calibrated SEC column, indicating that the protein in 5 M GdnHCl still has a partially compact structure. Even so, the MALS analysis of the eluted fractions exhibited the size of oligomeric species (~ 15 nm). Thus, it is possible that the observed oligomers are transient species formed by labile interactions that can be broken by shear forces between proteins and the chromatographic bed and later restructured upon elution. Contrastingly, the protein eluted from IMAC in 2 M GdnHCl eluted in the void volume (15.8 mL) and a few contiguous fractions in the SEC column (Fig. 3f), in agreement with the size expected for VLPs. This step also facilitated the imidazole elimination of the VLP fraction. The overall yield of SpyTag-VP2 VLPs obtained by this in-column folding and off-column self-assembly was 14.9 mg per L of culture media, comparable to the dialysis method (~ 40 mg/L) but saving at least 34 h per batch.

### Effect of the amount of protein loaded into the IMAC column on the species size obtained

**Fig. 4:**
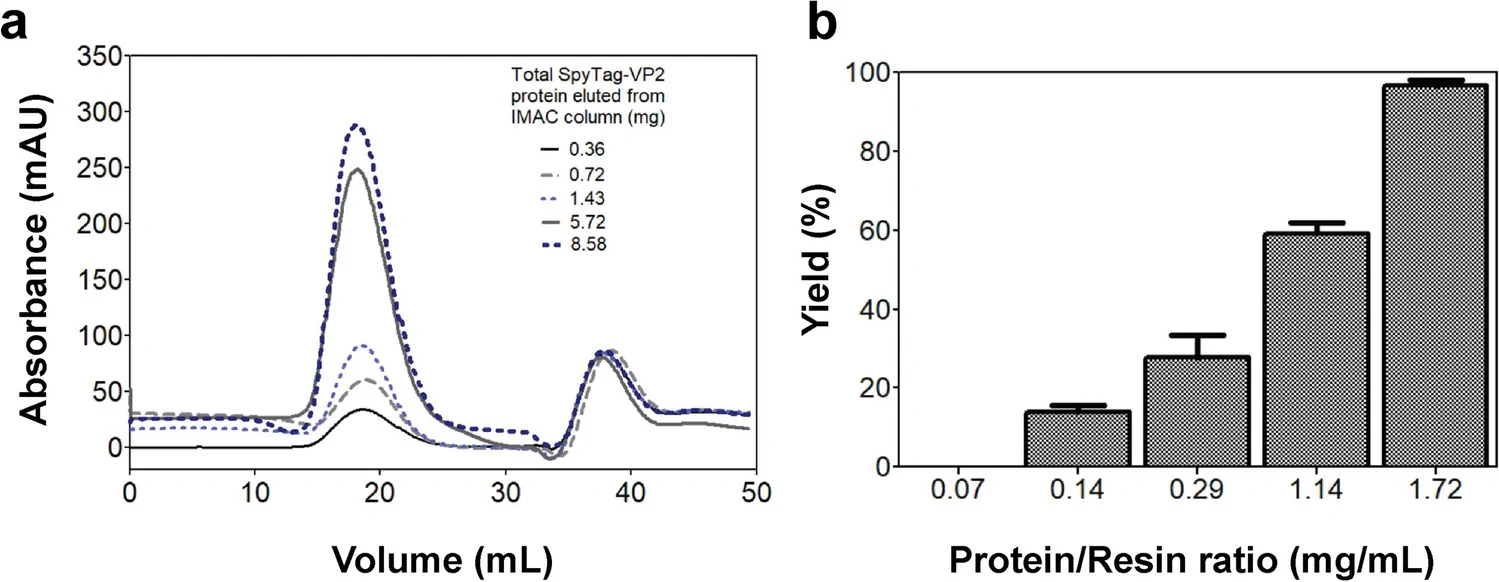
SEC elution profiles of SpyTag-VP2 loaded with different amounts of the SpyTag-VP2 peak obtained by IMAC (**a**). Yields of SpyTag-VP2 VLPs recovered from the IMAC column loaded with different protein amounts (**b**)

VLP assembly without nucleic acids is a concentration-sensitive process (Fuertes et al. 2023). Early observations of the association state of SpyTag-VP2 eluted from IMAC columns suggested that a low protein mass bound to the resin produces species with smaller diameters than those expected for a VLP. The systematic analysis of the effect of the amount of protein loaded into the IMAC column confirmed that the assembly of VLPs during the elution step is highly dependent on the protein mass (Fig. 4a). The MALS analysis of the fractions obtained from the size exclusion chromatography of the different elution peaks obtained from the IMAC column showed a range of species from 13 to 17 nm in diameter eluted at low protein mass, producing a poor VLP recovery. We observe that for quantitative recovery of VLPs, the mass of SpyTag-VP2 bound to the column has to be approximately 1.72 mg per mL of resin; above this concentration, approximately 95% of the protein loaded in the IMAC column was recovered in the form of VLPs after the SEC step (Fig. 4b). Thus, the VLP formation during the off-column assembly process is controlled by the amount of the loaded protein into the column, preventing the protein aggregation observed in the dialysis process when protein concentration is above 0.7 mg/mL.

### Functional analysis of the off-column assembled VLPs

**Fig. 5:**
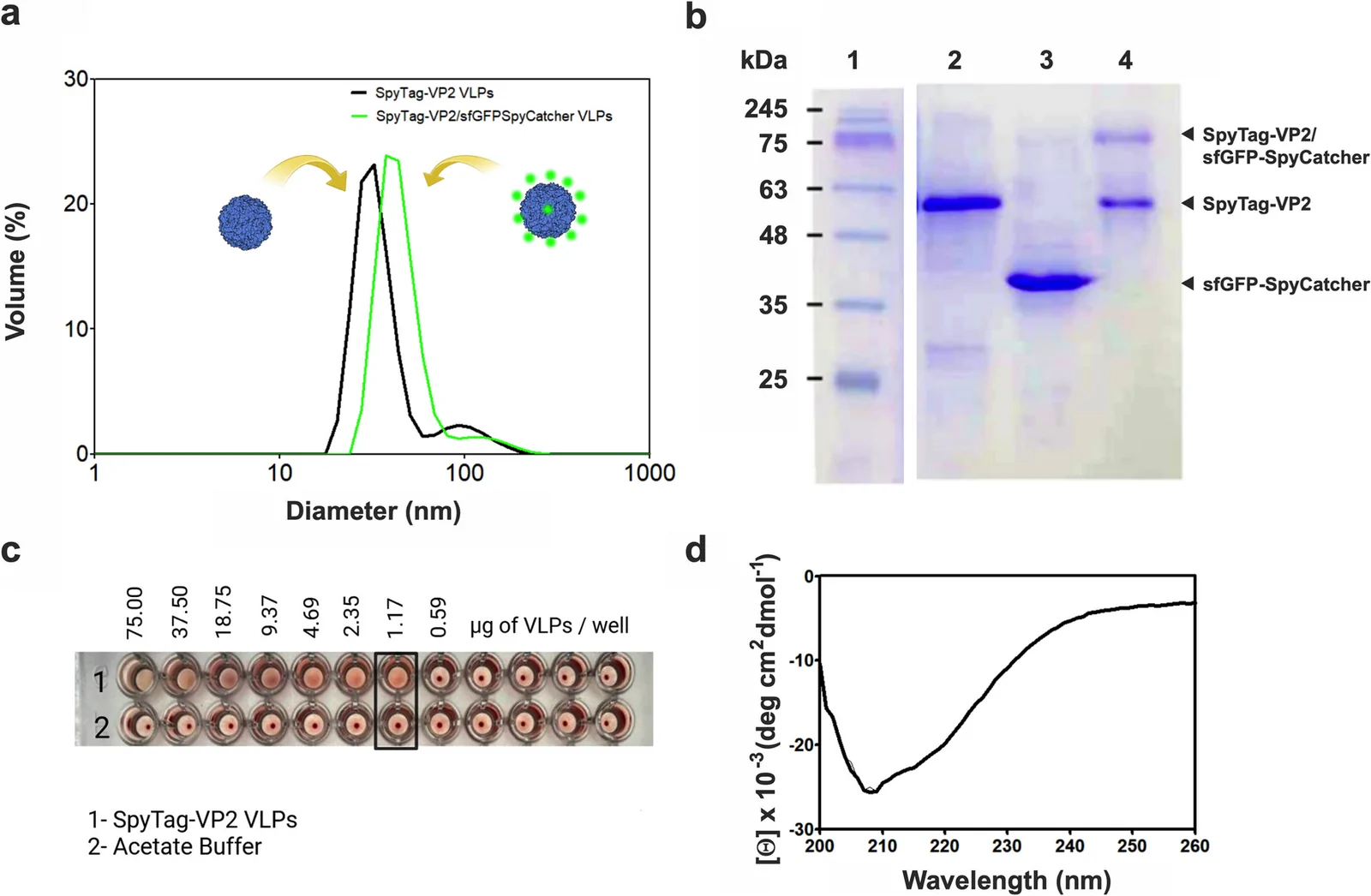
DLS analysis of the SpyTag-VP2 VLPs and the same particles decorated with sfGFP-SpyCatcher (**a**). SDS-PAGE analysis of SpyTag-VP2 protein and the particles decorated with the chimera sfGFP-SpyCatcher (**b**). Lane 1, molecular weight marker; lane 2, SpyTag-VP2 VLPs purified by SEC; lane 3, sfGFP-SpyCatcher; lane 4, reaction between SpyTag-VP2 VLPs and sfGFP-SpyCatcher producing a high-molecular-weight species of about 75 kDa. Hemagglutination test of SpyTag-VP2 VLPs assembled by the off-column method, row 1, SpyTag-VP2 VLPs; row 2, control buffer (**c**). CD spectrum of the SpyTag-VP2 VLPs assembled by the off-column method (**d**)

To confirm the functionality of the SpyTag peptide on the surface of the off-column assembled VLPs, we conjugated the SpyTag-VP2 VLPs with its bioorthogonal pair SpyCatcher fused to the sfGFP, sfGFP-SpyCatcher as described previously (Cayetano-Cruz et al. 2018). DLS analysis of SpyTag-VP2 VLPs before and after conjugation with sfGFP-SpyCatcher showed an apparent increase in size attributable to the bioconjugation (Fig. 5a). The sfGFP-SpyCatcher conjugation to SpyTag-VP2 VLPs was also demonstrated by SDS-PAGE analysis (Fig. 5b), in which a high molecular weight band appears after incubating both species for 1 h at room temperature in PBS 1.5 × added with L-Arg 0.4 M. Hemagglutination of RBCs was used to assess the correct assembly of VLPs produced by the off-column method. Hemagglutination occurs through the interaction of the globoside receptor from the cell’s surface with residues 339–404 of three different subunits located at the threefold symmetry axis on the B19V capsid (Huang et al. 2014). The SpyTag-VP2 VLP hemagglutination test resulted in a positive (Fig. 5c), while the negative control did not show agglutination and formed a red dot at the bottom of the well. A titer of 1:128 was obtained, corresponding to 1.1 × 10^6^ particles. Finally, the correct folding of the SpyTag-VP2 VLPs was also assayed by CD spectroscopy (Fig. 5d), which produced a spectrum similar to that reported by Sánchez-Rodríguez et al. (2012) for VP2 VLPs assembled in acetate buffer, confirming the correct folding of the protein.

## Discussion

The most common pattern capsid fold in non-enveloped viruses is an eight-strand antiparallel β-barrel. This canonical structural motif is found across various icosahedral viruses (Krupovič and Bamford 2008; San Martín and Van Raaij 2018). Also, it has been reported that some viruses can drive the formation of amyloid deposits during the infection period. These protein deposits are constituted mainly by fibrillar structures with a common cross-β supramolecular organization (Michiels et al. 2021; Nyström and Hammarström 2022). Thus, amyloid material and virus capsid proteins may share this structural pattern.

Besides, IBs were considered a waste product for a long time; nowadays, X-ray fiber diffraction and Fourier transform infrared spectroscopy (FTIR) analysis of IBs shown that these materials are often enriched in β-sheet secondary structure, suggesting an amyloid-like character instead of an amorphous protein deposit nature (Carrió et al. 2005; De Marco et al. 2019). Nonetheless, to make IBs a viable source of recombinant proteins, the most critical step is the conversion of the often inactive, insoluble, and misfolded IB proteins to their soluble bioactive form. Therefore, a major focus of IB process development is on the solubilization and renaturation steps. Even though long and cost-intensive downstream applications cause bottlenecks in IB-based processes, the overall time–space yield tends to be favorable for IB production compared to periplasmic or soluble protein production (Chen et al. 2018).

In this research, the protein SpyTag-VP2 was concentrated, purified, refolded, and assembled by IMAC. According to our results, SpyTag-VP2 IBs have an amyloid nature to a certain extent, which explains why B19V VP2 or its chimeras are so difficult to solubilize and require at least 5 M GndHCl, while 8 M urea cannot solubilize VP2 IBs (Sánchez-Rodríguez et al. 2012). Moreover, this unfolded protein undergoes protein–protein interactions when protein concentration is increased from 0.1 to 0.7 µM. This is not surprising as hydrophobic interactions emerge as a result of protein crowding. Nevertheless, it is unclear if such interactions are the same leading the monomer association to form the trimeric capsomers of B19V.

In this approach, once the SpyTag-VP2 is partially purified from the IBs in the form of denatured protein and re-bounded to the IMAC column, the critical concentration for VLP assembly is easily achieved due to the affinity tag that eases concentrating the protein in the chromatographic bed. Typically, denaturing agents keep the proteins from interacting with each other. However, the protein–protein proximity created by the immobilization in the IMAC matrix and the natural intersubunit contact degeneracy proper of the structural proteins of the virus may favor the formation of larger oligomeric structures, favoring the assembly of VLPs in a down-hill process during the elution step.

Here, VP2 and its chimeras are provided with a 6xHisTag located at their C-termini, and, according to the available structures (PDB codes 1S58 and 6NN3), the C-termini of VP2 are located on the internal surface of these particles. In agreement with this observation, self-assembled VLPs from VP2 and its chimeras do not bind to IMAC columns in our experiments. This result implies that SpyTag-VP2 will only assemble into VLPs once released from the column. In line with our hypothesis, self-assembly may occur off-column upon elution, either through the chromatographic bed or after leaving the column, when protein concentration is in the correct range and the appropriate pH and ionic strength conditions. When the elution step is carried out in the range of 2.5–4.5 M GdnHCl, the elution pattern is comparable to that of 5 M GdnHCl, with a reduced presence of oligomeric species. On the other hand, at a concentration of 2 M GdnHCl, the flowthrough from the IMAC column, previous to the elution step, has a significant amount of SpyTag-VP2. It is plausible that such protein is folded and forms oligocapsomeric species that impose steric hindrance to the HisTag present on the internal concave surface of these capsid fragments. This is the step in which most of the protein is lost, impacting the global yield of the procedure. Nevertheless, this protein can be recovered and recycled. Indeed, if a complete purification step at 5 M GdnHCl is carried out before the on-column refolding and off-column assembly, all the leaked protein from the latter step could be easily recovered and recycled. Moreover, if the off-column assembly occurs in the presence of pharmacologically active substances, it could be possible to load the VLPs with these compounds, producing a new vector for such drugs that can then be tagged to specific cells or tissues.

Several chromatographic procedures have been used to help in protein refolding (Wang et al. 2005). For example, the recombinant His-tagged apoaequorin was purified and refolded by IMAC (Glynou et al. 2003). The purification was carried out in a combination of batch and column chromatography. The cell lysate, containing 6 M urea as denaturant, was mixed with the chromatographic agarose and then shaken for 90 min at room temperature. The mixture was applied to a column, and urea was removed slowly with a 10 bed volumes gradient from 6 to 0 urea at 4 °C. In the last wash, with no urea, 5 bed volumes of 200 mM imidazole were used to elute the protein. A 2–4 molar excess of coelenterazine was added to reconstitute aequorin, and the reactivation reaction proceeded by 18–20 h at 4 °C. Another study also applied an artificial chaperone-assisted IMAC strategy to the widely used fluorescent probe protein EGFP (Dong et al. 2009). For binding to the IMAC column, the EGFP was complexed with cetyltrimethylammonium bromide (CTAB). Then, to promote EGFP refolding, CTAB was removed from the protein by capturing it with b-cyclodextrin (b-CD). The on-column refolding approach recovered 80% of the EGFP fluorescence. Off-column oligomerization has also been achieved with a trimeric membrane protein from chloroplast, the light-harvesting complex II (LHC2) (Rogl et al. 1998). LCH2 was expressed in *E. coli* as IBs, then resuspended with 8 M urea and bound to an IMAC column. The column was washed first with lithium dodecyl sulfate to remove lipidic components and then with the milder detergent octyl-glucoside. Finally, to promote the refolding of the protein, chlorophyll and carotenoids were applied to the column as the native form of LCH2 binds these pigments, and the excess pigments were washed with an adequate buffer. After elution with imidazole, the protein was folded and trimeric. According to the authors, trimerization presumably occurs as the monomers are released from the column, just as it is proposed here for SpyTag-VP2.

A different shade of the chemical definition of VLP components is the very nature of the participating proteins. Our group has demonstrated the co-assembly of different chimeras of VP2; for this purpose, VP2 and VP2 chimeras, generally carrying heterologous peptides or proteins fused at their N-termini, have been expressed in *E. coli* as IBs, then individually purified by IMAC under denaturing conditions, and finally, mixed in well-defined molar ratios to produce hybrid VLPs harboring the different proteins in each particle. Remarkably, the last step of refolding and self-assembly through equilibrium dialysis takes a minimum of 36 h, and for some chimeras, it may take up to 72 h (Cayetano-Cruz et al. 2018, 2019; Salazar-González et al. 2019; Jiménez-Chávez et al. 2021). However, using the off-column assembly approach reduces the time to obtain VLPs by at least 24 h and using only chromatographic steps. Also, given that the self-assembly of VLPs from IBs by the dialysis method has been successfully applied to another member of the Parvoviridae genus, the adeno-associated virus (AAV) (Le et al. 2019), it can be foreseen that off-column assembly approach may also be effective for other members of this genus.

Finally, the off-column assembled SpyTag-VP2 VLPs preserved the ability of the SpyTag peptide to bioconjugate with its orthologous pair, SpyCatcher, revealing that these particles are equivalent to those assembled by dialysis. This is of particular importance as it allows the construction of decorated VLPs for building biomaterials for different purposes, like antigen carriers for vaccine development, protein or peptide carriers for cell tagging and delivery, and as a scaffold for constructing multifunctional biomaterials harboring many of these properties.

Off-column self-assembly of VP2 chimeras is a fast new approach for B19V-VLPs manufacture through an easily scalable laboratory process, offering a scalable alternative to the conventional VLP assemble methods. Despite the simplicity of the process, this strategy has tremendous potential for pharmaceutical applications, like vaccine development and drug loading into the inner core of B19V VLPs at the off-column self-assembly stage of the manufacturing process.

## Data Availability

Data is available on reasonable request.
